# Analysis of User Satisfaction with Online Education Platforms in China during the COVID-19 Pandemic

**DOI:** 10.3390/healthcare8030200

**Published:** 2020-07-07

**Authors:** Tinggui Chen, Lijuan Peng, Xiaohua Yin, Jingtao Rong, Jianjun Yang, Guodong Cong

**Affiliations:** 1 School of Statistics and Mathematics, Zhejiang Gongshang University, Hangzhou 310018, China; Cherrylijuanpeng@163.com (L.P.); yinxh0213@163.com (X.Y.); rjt323@126.com (J.R.); 2 Department of Computer Science and Information Systems, University of North Georgia, Oakwood, GA 30566, USA; Jianjun.Yang@ung.edu; 3 School of Tourism and Urban-Rural Planning, Zhejiang Gongshang University, Hangzhou 310018, China; cgd@mail.zjgsu.edu.cn

**Keywords:** public health emergencies, online education platform, user satisfaction prediction, emotion mining

## Abstract

The outbreak of Corona Virus Disease 2019 (COVID-19) in various countries at the end of last year has transferred traditional face-to-face teaching to online education platforms, which directly affects the quality of education. Taking user satisfaction on online education platforms in China as the research object, this paper uses a questionnaire survey and web crawler to collect experience data of online and offline users, constructs a customer satisfaction index system by analyzing emotion and the existing literature for quantitative analysis, and builds aback propagation (BP) neural network model to forecast user satisfaction. The conclusion shows that users’ personal factors have no direct influence on user satisfaction, while platform availability has the greatest influence on user satisfaction. Finally, suggestions on improving the online education platform are given to escalate the level of online education during the COVID-19 pandemic, so as to promote the reform of information-based education.

## 1. Introduction

The global spread of COVID-19 resulted in the suspension of classes for more than 850 million students worldwide, disrupting the original teaching plans of schools in these countries and regions. Soon later, many countries started to offer online teaching to students by Zoom, Skype, FaceTime, etc. in order to promote online education and restore the normal teaching order, and on 6 February 2020, the Ministry of Education of the People’s Republic of China announced to vigorously support information-based education and teaching, and enhance the platform’s service capacity to support online teaching. In response to the outbreak of the epidemic, the online classroom has become a necessary way to maintain normal teaching order. Ding Ding, Fanya, and other office meeting software tools in China deliver services such as an online classroom and online teaching. However, these online education platforms have problems such as system jams and the inability to replay live broadcasts. It is necessary to study whether these network education platforms can meet the needs of teachers and students, whether the network teaching can complete the teaching tasks with high quality, whether the network education can become an effective means of special period education, and put forward suggestions to promote the development of network education according to the research results.

At present, scholars in various counties have carried out studies on online education platform evaluation, including using an analytic hierarchy process (AHP) and the partial least square method to establish the satisfaction evaluation system of online education platforms. For example, Wilbur [1] conducted a structured self-assessment and peer review using an instrument systematically devised according to Moore’s principles of transactional distance to evaluate the online component of a blended-learning degree program for pharmacists, and he found that a number of course elements for modification could enhance the structure, dialog, and autonomy of the student learning experience. Ryan et al. [2] reported the results of a pre-post-test questionnaire designed to evaluate the impact of the professional development intervention, and the analysis showed high scoring means with many items in the questionnaire statistically significant (*p* < 0.05, CI = 95%). Chiao et al. [3] constructed a virtual reality tour-guiding platform and 391 students from a technological university in Taiwan participated in the study. The results indicated their learning effectiveness and technology acceptance within the education system. However, these traditional methods have some shortcomings in the evaluation process, such as complex calculation and unreasonable weight determination. In addition, the main forms of online education in the past were watching public classes of famous universities and tutorial videos of institutions. However, during the epidemic period, online education is mainly in the form of class-based teaching by teachers of their own school, which is an extension of the original offline education. Previous studies on the satisfaction of online education platforms did not take the new factors brought by the epidemic into account, such as ease of use and quality of interaction.

Based on this, combined with the background of public health emergencies, this paper evaluates the online education platform in China from the perspective of students. First of all, the emotional analysis of online user comments was conducted to find out the factors affecting the satisfaction of online education platforms. Then, the satisfaction evaluation system was established on its basis. The index coefficient was determined by using the structural equation, and the back propagation (BP) neural network model was further used to predict the satisfaction of online education platforms.

The structure of this paper is organized as follows: Section 2 is a literature review. Section 3 collects and processes users’ online and offline data. Section 4 conducts emotional analysis of online comments. Section 5 carries out an empirical analysis of user satisfaction. Section 6 is the summary of the paper and the prospect of future work.

## 2. Literature Review

### 2.1. Online Education Platform

Many experts and scholars including Anderson [4] and Sultan [5] etc. from various counties have conducted research on online education with the vigorous development of the online education industry. Some typical studies are as follows: Chan et al. [6] described a novel technique combining Internet- and cloud-based methods to digitally augment the classic study group used by final-year residents studying for the Royal College of Physicians and Surgeons of Canada examination. Gofine and Clark [7] piloted the integration of Slack into their research team of one faculty member, one research coordinator, and approximately 20 research assistants. Statistics describing the app’s usage were calculated twelve months after its implementation and their results indicating heavy usage by both research professionals and assistants were presented. Thor et al. [8] investigated the impact of the online format on the discussion quality and the survey results showed that students preferred using Voice Thread for presenting, learning from other presentations, and discussing presentation content by performing this process in the classroom. Botelho et al. [9] assessed the usefulness, ease of use, ease of learning and satisfaction of a cloud-based clinical progression practice record when compared to a traditional paper practice record. The results suggested that a digital clinical book, using free cloud-based collaboration tools, was more useful, easier to use and learn from and more satisfactory than a traditional paper recording system. Chapman et al. [10] proposed four important dimensions of coverage, participation, quality and student achievement, and constructed a massive open online course (MOOC) quality assessment framework, helping MOOC organizations make a series of measures for monitoring and improving. Hrastinski [11] put forward a theory in his research: if we wanted to enhance online learning, we needed to enhance online learner participation. Miri and Gizell [12] showed in their research the need for rethinking the way conventional online ethics courses are developed and delivered; encouraging students to build confidence in learning from distance, engaging them in online active and interactive experiences. Anderson et al. [13] pointed out that healthcare professionals could share their expertise through online education and incorporate this teaching into their annual learning. Kamali and Kianmehr [14] pointed out that the public’s interest in online education was growing, while educational institutions’ interest in online education was going down. They held the view that in order to change the negative effect of online education, it was necessary to provide students with a suitable network environment, and discussed online education from the perspective of students. Alcorn et al. [15] evaluated satisfaction of online education from the number of class participants, the participation rate of homework, the completion rate and the improvement of grades. Asarbakhsh and Sars [16] believed that the broken-down system, failed video connection or unusable use affected user satisfaction. From the perspective of users and designs, David and Glore [17] pointed out visual content was quite important to improve participation and interaction of users. Based on the technology acceptance model and taking 172 online learning users as the objects, Roca et al. [18] analyzed online learning satisfaction. The results showed that the user’s online learning satisfaction was mainly determined by the user’s perception of the usefulness and quality of the course, the quality of the platform and the website service and the degree of expected achievement. Lin and Wang [19] believed that students’ satisfaction would be influenced by the difference of technology, the characteristics of teachers, students and courses. Panchenko [20] held the view that the MOOC teaching mode could develop teachers’ careers, improve teaching skills, and enable teachers to consider and examine their teaching activities from more perspectives. Kravvaris and Kermanidis [21] testified that social networks contributed to MOOC development. The literatures [22,23] found that learners’ autonomy played an important role in learning through the empirical study of MOOC. Through exploratory factor analysis (EFA) and confirmatory factor analysis (CFA), Parra-González and Segura-Robles [24] concluded that “game” was regarded as a motivating factor in the educational process, which could promote students to participate in the learning process more actively.

According to the above research results, many scholars study online education and establish many evaluation models. However, in the process of carrying out online education during this epidemic, many new problems arise in the new form of online education. This requires that new factors affecting user satisfaction be taken into account in the study. Based on this, this paper collects online user comment data to obtain the new factors affecting user satisfaction and establishes an evaluation system that can better reflect the satisfaction of online education platforms during the epidemic.

### 2.2. Customer Satisfaction

Customer satisfaction is the state of pleasure or disappointment formed by the comparison of the perceived effect of a product or service with the expected value. Previous scholars and experts have conducted many studies on customer satisfaction and established models, which can be divided into macro- and micro-models. Macro model: since the 1990s, many countries have carried out a national customer satisfaction index measurement work, regarding customer satisfaction index as a macroeconomic indicator to measure the customer satisfaction degree of a product or service. For instance, in 1989, Fornell [25] put forward the customer satisfaction index (CSI) by considering customer expectation, post-purchase perception and purchase value. Under the guidance of professor Fornell, based on the annual customer survey data of more than 100 enterprises over 32 industries, a Swedish Customer Satisfaction Barometer (SCSB) was constructed by using the Fornell model and calculation method. Under the guidance of Anderson and Fornell [26], America published the American Customer Satisfaction Index (ACSI) on the basis of the SCSB. The ACSI added perceived quality to measure the reliability of a product or service, as well as customer satisfaction. In 1992, Germany constructed the Deutche Kundenbarometer (DK) model, which consisted of 31 industries [27]. The European Union constructed the European Customer Satisfaction Index (ECSI) by adopting a comparative advantage over a wide variety of countries. This model omitted customer complaints but added company image, dividing perceived quality into perceived hardware quality and perceived software quality [28]. Micro model: the measurement model of customer satisfaction in micro fields is abundant. For instance, Tversky [29] put forward a variation model in 1969. Oliver [30] established a general model for measuring subjective inconsistencies in 1980. Sasser et al [31] proposed customer model with service level. Parasuram et al. [32] created the SERVQUAL scale to evaluate service quality. They divided the factors that determine service quality into five categories: reliability, responsiveness, assurance, empathy and tangibility.

From the above research outcomes, many scholars and institutions of various counties study the satisfaction evaluation system and establish many models. However, previous studies did not consider the impact of public health emergencies. On the basis of full reference to previous studies, this paper, in the context of the COVID-19 pandemic, optimizes the indicators used in previous studies and establishes a satisfaction evaluation model by considering the impact of public health emergencies.

## 3. Data Collection and Processing

In this paper, data are obtained through a questionnaire survey and web crawler. The online data obtained by web crawler technology are trustable and objective without restriction. Therefore, this paper uses the data obtained by web crawler to make a macro analysis of the user experience on the current online network teaching platform, and finally summarizes the main factors affecting the user experience satisfaction. Although the traditional questionnaire has many limitations, the obtained data are more targeted, diverse and abundant, which can test the ranking of impact factors summarized by the crawler data. Therefore, this paper combines the two methods to comprehensively acquire online and offline experience data of users.

### 3.1. Collecting Comments on Online Teaching Platforms

#### 3.1.1. Platform Selection

At present, there are a large number of online teaching platforms in China, such as MOOC, and Tencent Class. We are unable to assess all platforms. Thus, it is necessary to select representative platforms to evaluate. In this study, data samples of online education platforms were selected on ASO100 (a big data service platform for analyzing the App Store, Qimai, Beijing, China), and the ranking of the education category (updated on 17 April 2020) was screened based on the download volume, comments and popularity of the platform as the representative measurement criteria of the platform. The platform ranking results are presented in Table 1.

As illustrated in Table 1, in this study, Ding Ding (Alibaba, Hangzhou, China), Tencent Meeting (Tencent, Shenzhen, China), Tencent Class (Tencent, Shenzhen, China), Chaoxing Learning (Chaoxing, Beijing, China) and MOOC (Chaoxing, Beijing, China) were selected as the representative platforms for online teaching. These platforms have both synchronous and asynchronous learning capabilities, with no difference in system quality.

#### 3.1.2. Collecting Comment Data

In China, schools began to implement online teaching on 17 February 2020; therefore, this study collected comments on those teaching platforms from 17 February 2020 to 17 March 2020.

### 3.2. Questionnaire Data Collection and Processing

#### 3.2.1. Questionnaire Design

From the comments on ASO100, it is difficult to determine all the factors that affect an online teaching platform. To obtain a more targeted evaluation of user experience, this study adopted a questionnaire survey, whose targets were primary school, middle school, high school, university, and postgraduate students. By sorting and analyzing relevant literature, we designed the questionnaire with three parts, as demonstrated in Table 2.

In the second part, user experience satisfaction questions used a Likert scale. The scoring system was 1–5, where 5 represented strong agreement and 1 represented strong disagreement. The higher the score was, the more strongly the respondents agreed with the statement.

#### 3.2.2. Questionnaire Validity Test

During the epidemic period, the questionnaire tool named Wenjuanxing was used to collect information. After investigation, a total of 800 questionnaires were received, with 712remainingafter the removal of invalid questionnaires. After data collection, 712 questionnaires were coded and entered into SPSS statistical software (SPSS Statistics 25.0 HF001 IF007, IBM, Armonk, NY, USA) to perform descriptive analysis, and reliability and validity analysis.

##### Reliability Test

The reliability test, which measures data reliability, is used to test the stability and consistency of questionnaire data. In this study, Cronbach’s *α* was used to test the internal consistency of the questionnaire data, whose coefficient was between 0 and 1. In general, a coefficient greater than 0.7 indicates that the questionnaire can passes the internal consistency test. In contrast, a coefficient less than 0.7 indicates that some questions must be discarded. The reliability test results are presented in Table 3. In this questionnaire, six Cronbach’s *α* coefficients were all greater than 0.7, indicating that the internal reliability of each first-level indicator of the questionnaire was high.

##### Validity Test

The validity test can be divided into content validity and structure validity. The questions in this questionnaire scale used relevant literature for reference to ensure high content validity. The structure validity passed the KMO (Kaiser–Meyer–Olkin) test and the Bartlett test. Generally, when KMO is greater than 0.5, and the significance level of the Bartlett test meets the significance requirement of a two-tailed test, it is considered that the questionnaire passes the validity test. The results of the validity test are presented in Table 4. It can be seen that the test values of the KMO and Bartlett test of the six first-level indicators in the questionnaire all met the requirements, indicating that they passed the validity test.

#### 3.2.3. Data Analysis

**Figure 1:**
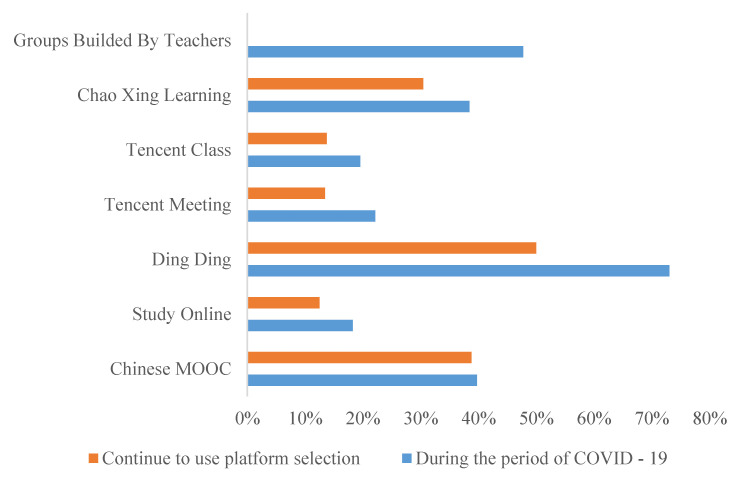
Choice of online teaching platform.

Referring to Bawa’s method of data analysis which includes descriptive statistics, analysis of variance (ANOVA) and T-tests [33], this paper analyzes the questionnaire data as follows: In this questionnaire survey, 26.6% of respondents were male while 73.4% of respondents were female. The majority of the participants were middle and high school students, junior college students, undergraduate students, and graduate students. Primary school students may produce invalid questionnaires due to their difficulties in text comprehension. According to the survey on the terminal types of online teaching platforms used by participants, mobile phones accounted for 84.62%, followed by laptop computers and tablet computers. The key questions in the questionnaire were analyzed to understand the data characteristics, as illustrated in Figure 1.

As can be seen from Figure 1, during the epidemic period, teachers mainly taught online using Ding Ding and self-established social groups (such as QQ group and WeChat group). As work management software, Ding Ding added on online teaching function in a timely manner in view of the epidemic. The results demonstrate that more than 50% of users continued using Ding Ding as an online learning platform after the epidemic ended.

**Figure 2:**
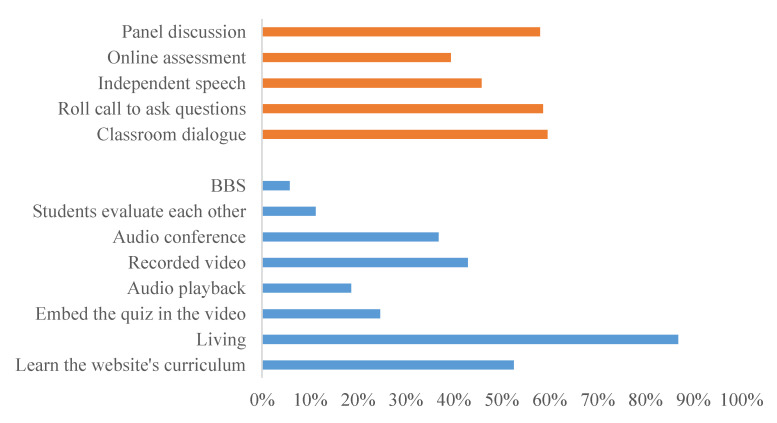
Online teaching and interaction.

As can be seen from Figure 2, most of the online teaching platforms can provide five learning modes and eight online interactive modes, which can effectively meet the existing teaching needs and provide feedback at any time. The two main teaching methods are online live broadcasting and existing courses on the platform. The MOOC platform contains rich teaching resources and has thus been favored and used as an online education platform for a long time.

**Figure 3:**
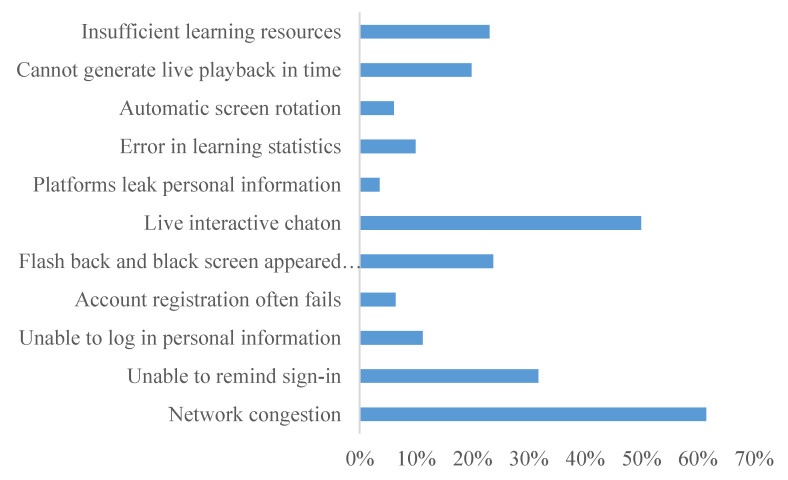
The use of online teaching platform.

As can be seen from Figure 3, there are 11 types of common problems regarding online teaching and courses that can be attributed to the problems mentioned in online comments, such as “network congestion”, “live interactive stuck” and “unable to log in personal information”. Therefore, to improve these issues, the five online teaching platforms can begin by addressing their live broadcast functions, system quality, and capacity enhancement.

## 4. User Emotion Analysis

The relationship between tutoring work and student emotions is of great significance to the cognitive re-evaluation of students. All comments were divided into different topics through data processing, and the key content in the comments was observed. In this study, the ROST (Regional Operations Support Team) [34] software was used to divide the emotional tendencies into three critical sets: positive, neutral and negative. Because the emotion dictionary of ROST is limited, the NLPIR-Parser(Natural Language Processing and Information Retrieval) [35] was used to score the emotion, which can be divided into the total score, positive score and negative score, to identify the platforms with better user experience. Using word frequency analysis, the advantages and disadvantages of platforms were extracted based on good or poor user experience.

### 4.1. Emotional Comment Analysis Based on ROST CM5.8.0

#### 4.1.1. Comments Analysis

It was shown that those students who carried out activities related to their emotions and the improvement of coexistence in tutoring had a greater cognitive reevaluation. Therefore, this paper makes an emotional analysis of user comments [36]. Based on the analysis results of ROST, this study integrated the positive, neutral and negative comments of the five platforms, as illustrated in Table 5.

Analysis of the positive, neutral, and negative comments indicated that Ding Ding and Tencent Class had more positive comments than negative comments, while Tencent Meetings, Chaoxing Learning, and Chinese MOOC demonstrated the opposite trend. In particular, Chaoxing Learning, and Chinese MOOC had more negative comments than positive comments.

#### 4.1.2. Analysis of Visualization for Semantic Network

**Figure 4:**
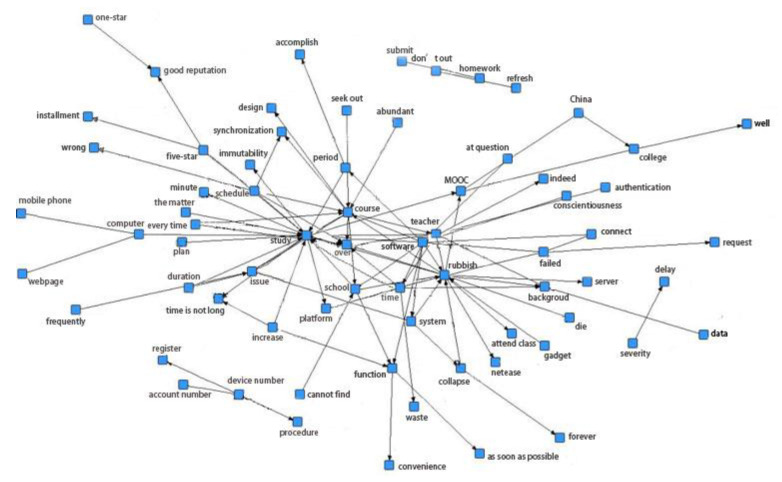
MOOC semantic network relationship.

A semantic network expresses the structure of human knowledge through the network. It is composed of nodes and arcs among the nodes. Nodes stand for concepts (e.g., events, things), while arcs represent the relationship between them. Mathematically, a semantic network is a directed graph, corresponding to a logical representation. In this study, the semantic network relationship diagrams of the five platforms were obtained through ROST analysis. A partial image of the MOOC semantic network relationship is presented in Figure 4.

(1) According to the semantic network relationship graph of Ding Ding, we use “study” as a node, and keywords that are close to this node are “epidemic” and “convenience”. This is because during the epidemic, Ding Ding expanded the educational function on its platform, enabling colleges to use it as an online teaching platform. Taking “software” as a node, a closer keyword is “live broad cast”, which also reflects that the teaching method of Ding Ding is mainly a live broadcast rather than students watching videos on their own. This method also increases the interactivity of online teaching, better mobilizes the learning atmosphere, and improves the quality of teaching. At the same time, due to the network congestion and negative user experience, most users gave a one-star rating to Ding Ding. This was explained by a Ding Ding official in time, leading to the popularity of the topic of “five-star payment by installment” on Weibo. It was due to the timely response that more users gave a five-star rating.
(2) According to the semantic network relationship graph of Tencent Meeting, we see that “five-star” and “meeting” are important nodes for Tencent Meeting evaluation. Taking “five-star” as a node, close keywords are “good reputation” and “epidemic”, indicating that during the epidemic, users felt positively about the platform. Taking “meeting” as a node, the keywords are “convenience” and “screen”, indicating that the Tencent Meeting platform was more convenient to use, and the quality and manner of screen presentation will affect the user experience.
(3) According to the semantic network relationship graph of Tencent Class, “teacher”, “software” and “class” were important nodes, and close keywords were “epidemic”, “attend class” and “many problems”, indicating that users valued the attendance function of Tencent Class, however, there are also several problems.
(4) According to the semantic network relationship graph of Chaoxing Learning, “software”, “rubbish”, “study”, and “course” were important nodes, and closer keywords were “submit”, “server”, “login”, “collapse” which reflected the many problems that occurred in the Chaoxing Learning, such as server crashes, inability to log in, and inability to submit the learning duration, which all had a negative impact on the user experience.
(5) According to the semantic network relationship graph of MOOC, “learning”, “rubbish”, “course”, and “software” were important nodes, and close keywords were “failed”, “connect”, “period”, “server”, and “progress”. From these nodes, we can see that the MOOC platform often failed to connect, the learning time could not be submitted, and the server crashed. The independent nodes “account” and “homework” indicate that the platform was unable to register an account, could not submit a job, and could not refresh. “Delay” and “severity” indicate that the delay in the MOOC platform was quite significant. “College” and “abundant” reflect that MOOC users were mainly college students, and the course types were abundant due to the characteristics of the MOOC platform. MOOC focuses on video teaching and conducts self-study courses, which are the primary reasons for its use.

Based on the analysis of the semantic network relationship graph obtained above, it can be seen that “epidemic”, “student”, “software”, “teacher”, “study”, “five-star” and “every time” were important nodes that appeared together in the five platforms. The closer the nodes are to the words, the closer their relationship is. The presence of “every time” and “five-star” was caused by the timely response to problems in Ding Ding, thus indicating the large influence of the official Ding Ding group.

### 4.2. Emotion Analysis Based on NLPIR-Parser

NLPIR emotion analysis mainly uses two technologies. The first is the automatic recognition of emotion words and the automatic calculation of weights. The co-occurrence relationship and bootstrapping strategy is adopted to repeatedly produce new emotion words and weights. The second technology is a deep neural network for emotion discrimination. Based on a deep neural network, the extended calculation of emotion words is performed, which is integrated into the final result.

By analyzing the comment data of the online teaching platforms, the emotion scores of the five teaching platform reviews were obtained, including the total emotion score, positive score, and negative score, as displayed in Table 6.

From the above emotion scores, it can be seen that the total emotion scores of Ding Ding, Tencent Meeting, and Tencent Class were all positive, while the total emotion scores of Chaoxing Learning and MOOC were negative, indicating that Ding Ding, Tencent Meeting and Tencent Class provided good user experience. In addition, the shortcomings of Chaoxing Learning and MOOC were more evident, as these platforms were not satisfactory for users. Because the negative score of Chaoxing Learning was much lower than the positive score, it is important to analyze the problems in the Chaoxing Learning platform to propose corresponding improvement measures.

### 4.3. Semantic Association Expansion

NLPIR adopts POS-CBOW (Problem Oriented System, Continuous Bag of Words), integrating the distribution characteristics of speech and words, using the word2vectormodel to train educational corpora, and automatically extracting semantic association relations.

**Figure 5:**
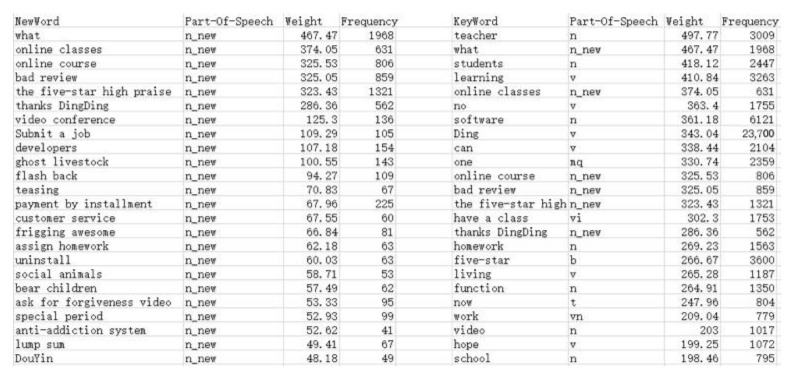
Comparison between new word and key word.

This paper expands the relevant semantics of high-frequency words on Ding Ding, Tencent Meeting, Tencent Class, Chaoxing Learning and MOOC. In addition, it captures new words and keywords with higher weight, and summarizes the factors that affect user experience. The part of the semantic graph related to Ding Ding is presented in Figure 5.

According to the relevant semantic expansion of the five platforms, the following words and phrases had the highest weight and the most frequent occurrence: “flash back”, “convenient and swift”, “customer service”, “projection screen”, “peep screen”, “horizontal screen”, “pop-up windows”, “staff service”, “prevention and control”, “interactive panel”, “dark mode”, “abnormal network”, “mobile office”, “Ding mail”, “call the camera”, “bundled software”, “shared screen”, “client end”, “verification code”, “vertical screen” and “network anomaly”, “recording”, “web version”, “screen recording”, “no privacy”, “blocking sight”, “rotating the screen”, “black screen”, “background playback”, “failed to load”, “scan code”, “system halted”, “submit a job”, “close microphone”, “network fluctuations”, “gesture check-in”, “personal information”, “submit homework”, “main interface experience”, “incompatibility”, “lost connection”, “self-rotating screen” and “mobile end”.

By classifying the new words and phrases mentioned above, we summarize the influencing factors that affect user experience, namely platform suitability, platform service type, platform privacy, platform teaching type, platform functionality, platform design environment, and network technology environment. By summarizing the factors influencing user experience for online teaching platforms during the epidemic, we determine the following Table 7.

By classifying the new words mentioned above, we summarize the influencing factors that affect user experience, namely, platform suitability, platform service type, platform privacy, platform teaching type, platform functionality, platform design environment, and network technology environment. By summarizing the factors influencing user experience for online teaching platforms during the epidemic, we can determine the following:

(1) The design environment of the platform should be more concise and easy to operate, and additional modes should be designed for different users at different times. For example, a “dark mode” at night can have better protective effect on the eyesight of students.
(2) At present, the types of electronic devices continue to rise. To expand the use of the platform, it is necessary to increase the development of each port of the tablet. In addition, to make students more comfortable during an online class, the platform should be able to adjust the horizontal and vertical screen any time.
(3) To improve the utilization and popularity of online teaching education platforms, customer services are essential. In the use of the platform, online customer service should always be available to address problems to prevent the wasting of learning time.
(4) During the epidemic, not only college students and graduate students, but also primary and secondary school students, must study online. However, the concentration abilities of the latter groups are relatively limited, therefore, teachers cannot blindly teach by rote and lecturing, but must use a variety of different methods, such as “you ask me to answer”, “face to face”, “students record learning videos”, and “real-time lecture”. The platform should enhance the type of functions and improve the quality of interactive devices while setting software functions.
(5) A stable network technology environment is the most important basis for improving teaching quality. If “network congestion” or “flash back” often occur in the use of the platform, the user experience as well as the usage rate will decrease accordingly.

## 5. Empirical Research on User Satisfaction

### 5.1. Building aUser Experience Satisfaction Index System

Based on the factors influencing user experience obtained by emotion, the advantages and disadvantages of online education noted by the users in the questionnaire (as illustrated in Figure 1, Figure 2 and Figure 3), and a large number of documents, this study aims to establish an effective but non-redundant index system. It combines Webqual 4.0 (availability, information quality, interaction quality) and the D&M (DeLone and McLean) system success model (information quality, system quality, service quality) to refine the influencing indicators. The indicators at each level correspond to the questions in the questionnaire. Among them, information quality and system quality are expressed together with subjective multiple choice questions, while others are expressed on Likert scales, as illustrated in Table 8.

### 5.2. Structural Equation Model

Structural equation modeling (SEM) is a common method to solve complex multivariable problems in social sciences. For example, in research fields such as social science, it is sometimes necessary to explore the relationship between more than one dependent variable and the influence path between hidden variables that cannot be directly measured. SEM can estimate abstract hidden variables through observable variables [37].

According to the above user satisfaction indicators, this paper uses the structural equation model to build the indicator system model and obtains the influence path coefficient of the latent variables on user satisfaction to draw the conclusion that the effects on user experience satisfaction weights are different. By using path analysis for the structural equation to determine the correlation between the indicators, and by decreasing the number of indicators to avoid redundant indicator construction, suggestions for improving the main influencing factors are proposed.

The IS (information systems) success model proposed by DeLone and McLean [38] measured user satisfaction on a website in terms of the service quality. McKnight and Chervany [39] constructed the factors influencing customer belief and supplier intention from the perspective of psychology and sociology, and each structure was further decomposed into two to four measures. Lao et al. [40] used text mining technology to establish a curriculum quality evaluation model that included five first-level indicators: curriculum content, instructional design, interface design, media technology, and curriculum management to provide a base standard for learners to evaluate the quality of the curriculum. Huang et al. [41] constructed an overall evaluation index system based on online education using four primary indices: system structure, educational resources, interactive mode, and market environment.

Based on the above analysis, this paper examines the factors influencing user satisfaction with the continuous usage of the intention of online teaching platforms by examining the four aspects of interaction quality, service quality, availability, and personal factors, and proposes the following hypotheses:

**Hypothesis** **1.** The interactive quality of the online teaching platform has a significantly positive influence on user satisfaction.

**Hypothesis** **2.** The service quality of the online teaching platform has a significantly positive influence on user satisfaction.

**Hypothesis** **3.** The availability of the online teaching platform has a significantly positive influence on user satisfaction.

**Hypothesis** **4.** The personal factor of the online teaching platform has a significantly negative influence on user satisfaction.

**Hypothesis** **5.** The user satisfaction with the online teaching platform has a significantly positive influence on the user’s willingness to continue using the platform.

#### 5.2.1. Model Estimation and Significance Test of Parameters

**Figure 6:**
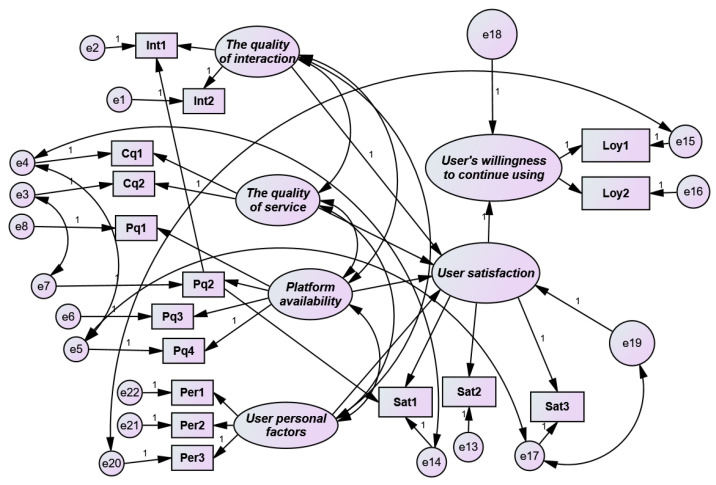
Parameter estimation of the model.

A structural equation model can effectively deal with the relationship of latent variables in the theoretical model of user satisfaction of an online network teaching platform. In this study, AMOS (Analysis of Moment Structures, IBM, Armonk, NY, USA) software was used to empirically study the structural equation model. On the premise that the reliability and validity analysis of the sample data met the requirements, parameter estimation of the model was performed based on the influencing factors of user satisfaction established previously. The estimated results of the model are presented in Figure 6.

The parameter analysis results of the initial model are listed in Table 9. After estimating the initial model, the significance test of the path coefficient and load coefficient was required. The “C.R.” (critical ratio) value was obtained by the disparity between the estimated parameter and standard parameter. When the absolute value of “C.R.” was greater than 1.96 and the corresponding probability *P* value was less than 0.05, it can be stated that there was a significant difference between the path coefficient and the estimated parameter value of 0 at 95% confidence. Therefore, it is assumed that the influence of the path coefficient was significant. It can be seen that the path coefficient of “user personal factors” on user satisfaction was unable to pass the significance test.

#### 5.2.2. Modified Structural Equation Model

After the structural equation model was completed, it was used to test the fitness degree of the sample data and perform model path analysis by calculating the fitness effect parameters. In AMOS, there are three evaluation indices for the fitness degree of a model: the absolute fitness index, value-added fitness index, and simple fitness index. Common fitness indices include *χ*^2^ (degree of freedom ratio) and GFI (goodness-of-fit index), AGFI (adjusted goodness-of-fit index), NFI (Normed fit index), RMSEA (Root Mean Square Error of Approximation), and CFI (comparative fit index). In this study, several common fitness indices were selected from the three fitness indices, and the calculation results are presented in Table 10.

**Figure 7:**
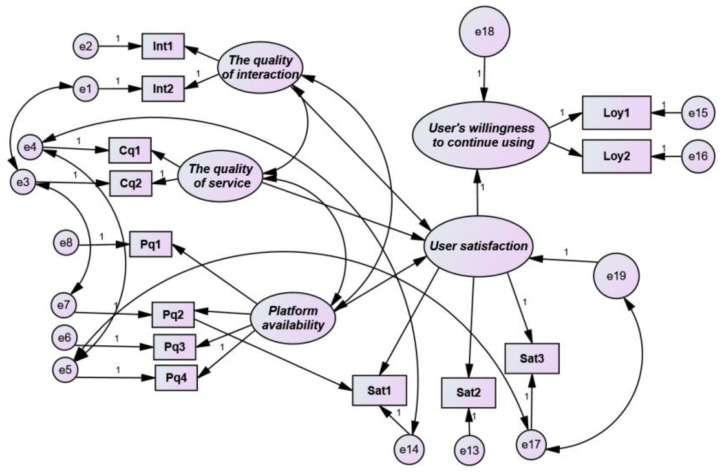
Modified results.

It can be seen that after deleting the path of “user personal factors,” all fitness indices were improved after the model was modified. According to the calculation results of all fitness indices, all reached the fitness standard of the model. The modified model is presented in Figure 7.

#### 5.2.3. Results Analysis

By modifying and testing the structural equation model and studying the sequence proposed by the research hypothesis, the path coefficients of the influencing factors are summarized in Table 11.

From the above analysis, we draw the conclusion that among the four major factors, personal factors had no direct influence on user satisfaction, indicating that users had a fair attitude and were not emotionally biased. Instead, platform availability had the largest influence on user satisfaction. In terms of availability, the function design and reasonable operation of the online teaching platform were the most important problems for users. In terms of interaction quality, the feedback for the homework assigned by teachers was the main factor affecting the sense of interaction experience. The influence of service quality on user satisfaction was mainly caused by matters such as timely response to problems, diverse course types, and learning extension. Users mainly hoped that the platform could meet their learning needs and provide necessary functions for learning; however, they did not have high expectations for the interface design of the platform. The correlation between the overall interaction quality, service quality, and availability was not high, indicating that the influence on user satisfaction was not high and that the construction of the structural equation was reasonable.

### 5.3. Predicted Satisfaction Model Based on the BP Neural Network

According to the above structural equation model results, the influence index of user satisfaction mainly involved platform availability, interaction quality, and service quality; personal factors had no direct effect on satisfaction. Therefore, this paper only regards the first three key indicators as input nodes and the degree of satisfaction as the output node by using the BP neural network model to forecast the degree of satisfaction.

#### 5.3.1. Overview of the BP Neural Network Algorithm

**Figure 8:**
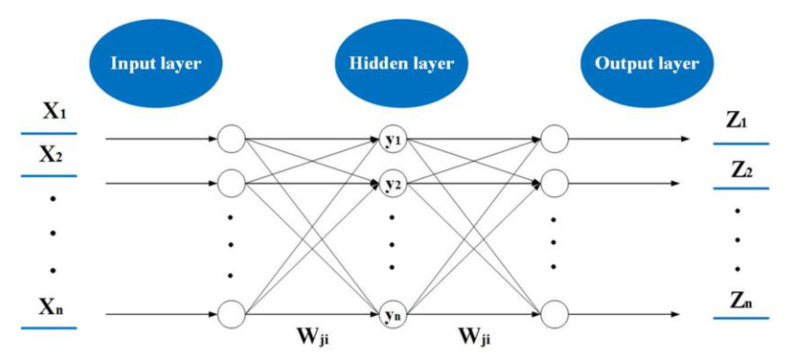
BP neural network structure.

A BP neural network [42], a type of artificial neural network, is a multi-layer network with one-way propagation, which is widely used in many fields such as public opinion [43,44,45], personalized recommendations [46], health monitoring [47], social network [48], feature subset selection [49,50] and emergency logistics [51,52]. A typical neural network consists of three layers: an input layer, hidden layer, and output layer. Each layer is composed of multiple neurons that are connected to each other by the weight coefficient; however, each neuron in the same layer is independent. The structure of a BP neural network is presented in Figure 8.

In the Figure 8, $X_{1},X_{2}\cdots X_{n}$ is the input layer neuron, $y_{1},y_{2}\cdots y_{n}$ is the activation function, $W_{ji}$ is the weight connecting the input layer, hidden layer and output layer, $W_{1},W_{2}\cdots W_{n}$ is the output layer neuron. The working principle of a BP neural network includes forward-propagation and back-propagation processes. Forward-propagation mainly refers to the input of training samples from the input layer, and the result of the output layer can be obtained through the connection of the weight coefficient between neurons and the activation function. If the prediction results of the output layer are not satisfactory, the back-propagation process is activated. The error signals of each unit are returned layer by layer along the original neuron connection pathway, and the weight coefficients of the neurons between layers are adjusted by calculating the error value. This training is repeated several times according to the training function until the network output error reaches a predetermined accuracy or the training times reach the set maximum iteration times. The algorithm steps are as follows:

Step 1: Data normalization;

Step 2: Data classification, extraction of normal training data, and data testing;

Step 3: Establishment of the neural network, including setting the number of nodes in each layer, activation function, etc.;

Step 4: Specifying parameters for training;

Step 5: Using training results, inputting test data after completing the training;

Step 6: Data anti-normalization;

Step 7: Error analysis, drawing, etc.

#### 5.3.2. Data Processing of the Predicted Model

Because each key index of the input layer corresponds to two or three questions in the questionnaire, each index actually corresponds to two or three data points. To obtain the index data of the input layer, the average value of the actual measured data in the questionnaire is used as the actual score data of this index in this paper. The actual scoring principle is as follows:

(1) $$X=\frac{\sum_{i=1}^{n}s_{i}}{n}$$

where *n* represents the number of questions corresponding to the key indicator and *s_i_* represents the score of the *i*th question corresponding to the key indicator. With this formula, the data of each node in the input layer of the BP neural network can be obtained.

In addition, the satisfaction results obtained from the questionnaire survey are hierarchical data, while the data in the BP neural network are normalized. As the result, the satisfaction data predicted by the output layer may not be classified. Therefore, this study used the existing literature [53] to classify the satisfaction level. The specific division is shown in Table 12.

#### 5.3.3. Implementation and Evaluation of Model

In this study, after several simulation experiments to compare the prediction effects, tansig was selected as the spread function of the input layer and hidden layer, purelin was selected as the spread function of the output layer, traingdx (self-adjusting learning efficiency method) was selected as the training function of the BP neural network (whose number of nodes in the hidden layer was set to six), and the maximum number of iterations epochs was 20,000.

**Figure 9:**
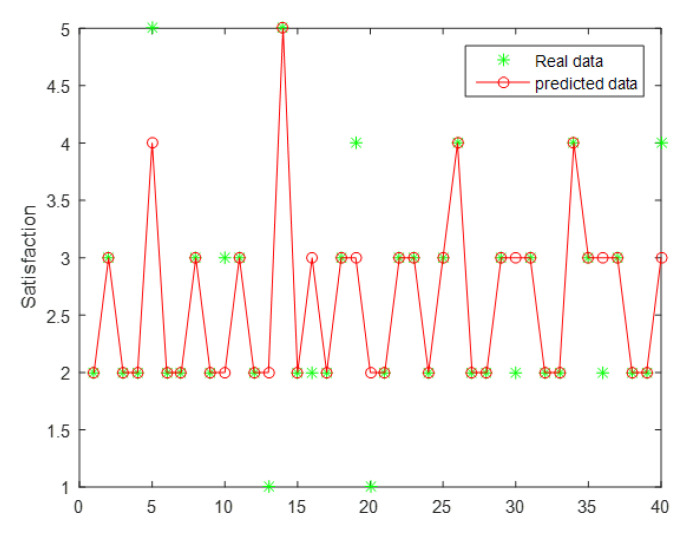
Predicted results.

The key indicator data were divided into a training group and test group, and the predicted results were obtained through training the set parameters, as illustrated in Figure 9.

As seen from Figure 9, the satisfaction level is mainly distributed in level 2 and level 3, indicating that the majority of users were dissatisfied with the existing online teaching platforms. Therefore, user satisfaction must be improved, and this study can be of great practical significance. In addition, the prediction results were generally accurate, but the prediction accuracy was only 77.5%, and the prediction effect was moderate, which may be due to the lack of training data in this study. If the proposed system is widely used in online education platform satisfaction prediction, the sample size can be increased, and the prediction accuracy may increase.

**Figure 10:**
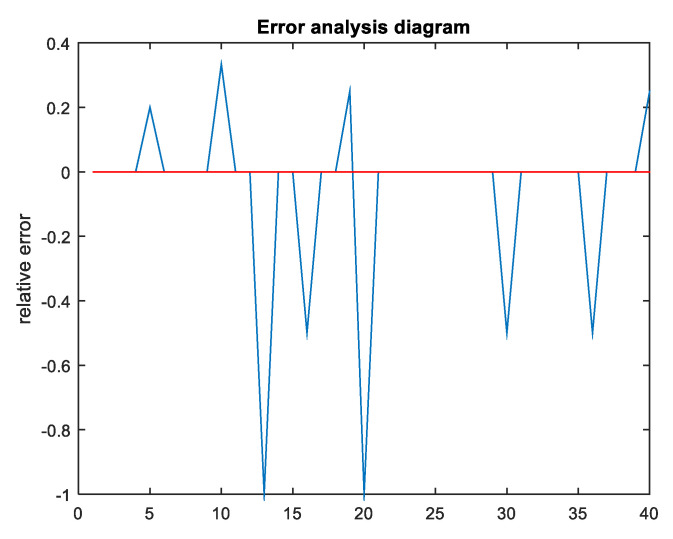
Error analysis diagram.

In addition, error analysis was performed on the prediction results of the BP neural network, and the results are presented in Figure 10.

As can be seen from Figure 10, the MSE only fluctuated in the range (−0.5, 0.4) with a small error value, indicating that the satisfaction prediction model established in this paper is effective. Other error index data are provided in Table 13.

## 6. Discussion

In this section, we will discuss four issues. First of all, in order to summarize the outstanding contributions of this paper based on the previous literature, we compared the conclusions of relevant literatures and discuss the relationship between the existing literatures and this paper. Secondly, in order to highlight the unique factors that affect the user satisfaction of online education platforms during the COVID-19 pandemic, we discussed the differences between online education during the COVID-19 pandemic and general online education, which is also the outstanding contribution of this paper. Once more, in order to solve the problem of capital investment in platform availability, we discussed several financing methods from the perspective of online education platform enterprises, and put forward feasible financing suggestions. Finally, in order to show that the conclusion of this paper can be applied to other countries in the world, we discussed the internationalization of conclusions.

### 6.1. Relationship with the Existing Literature

It is a priority to discuss the connections with other research on the topic. Previous studies, such as literature [16] and literature [18], focused on the satisfaction of users’ normal use of online education platforms when there was no public health disaster such as COVID-19. The influencing factors mainly include system fluency, visual content, curriculum usefulness, etc. The influencing factors considered in this paper are more combined with the problems in the online education teaching process during the COVID-19 pandemic, and the influencing factors such as platform availability, interaction quality, information quality, system quality, service quality and user personal factors are put forward. After structural equation analysis, it is found that platform availability is the most important factor, while user personal factors have little effect on satisfaction.

We will discuss it based on the relevant models mentioned in the above literatures. There are two basic antecedents of customer satisfaction in the SCSB model: expected quality and perceived value. User satisfaction is determined by the difference between perceived value and expected quality. Customer complaint and customer loyalty are outcome variables of customer satisfaction. The ACSI model is based on the SCSB model. The main innovation of the ACSI model is its ability to increase the quality of perception. The ECSI model inherits the basic structure and some core concepts of ACSI model, such as user expectation, perceived quality, perceived value, user satisfaction and user loyalty. Compared with the ACSI model, the ECSI model firstly removes the potential variable of user complaint in the ACSI model. Secondly, the ECSI model adds another potential variable, enterprise image, and divides the perceived quality into the quality evaluation of hardware and software.

The model used in this paper is constructed after referring to the above model, and combines emotional analysis and structural equation analysis. In the emotional analysis, we found that the factors that affect the satisfaction of online education platforms were platform availability, interaction quality, information quality, system quality, service quality and user personal factors. Combined with the relevant literatures, the platform availability, interaction quality, service quality and user personal factors are selected. Furthermore, after the structural equation analysis, the user’s personal factors are eliminated. Thus, in the model of this paper, the platform availability, interaction quality and service quality are the three major factors that determine user satisfaction. User satisfaction then affects the user’s willingness to continuously use the platform.

All in all, compared with the traditional SCSB, ACSI and ECSI models, the model constructed in this paper can better reflect the characteristics of an online education platform during the COVID-19 pandemic and, thus, it can better reflect the satisfaction of an online education platform.

### 6.2. Characteristics of Online Educationduring the COVID-19 Pandemic

In the past, teachers and students individualized and chose online education in a small range. At present, it has become a necessary choice for everyone. The seemingly helpless choice provides an opportunity for people to re-examine online education. Therefore, based on the COVID-19 situation, this paper studies the user satisfaction of online education platforms, which is different from the focus of user satisfaction under ordinary circumstances. When using an online education platform without COVID-19, more attention should be paid to the characteristics of the platform and the diversity of learning materials. According to the conclusion of this paper, the availability of the platform during the COVID-19 situation is the main factor affecting user satisfaction, which also reflects that users focus more on mobile terminal equipment, platform load, technology proficiency and other aspects. This study not only summarizes the weaknesses and constraints exposed by the online education platform during the COVID-19 pandemic, but also makes a significant contribution to the upgrading and optimization of online education, improving people’s cognition of online education, and increasing the acceptance and satisfaction of online education.

Due to the significant social impact of COVID-19, learners will have emotional problems at different levels due to the influence of home isolation and other factors, thus affecting the effectiveness of online learning. Therefore, educators are more concerned about the emotional changes of students in this unique period and put the physical and mental health of students first. Therefore, this study conducts relevant emotional analysis on online user comments. Additionally, the emotional factors caused by external factors were added to the study of user satisfaction. This is also different from other studies.

In the past, the teachers who provided courses on the online education platform system were specially trained by enterprises and only taught for a certain type of course. However, this COVID-19 outbreak is sudden. Teachers who use the online education platform are transformed into ordinary offline teachers. The preparation time of ordinary teachers is not enough. In addition, a considerable number of teachers lack sufficient knowledge of information technology, so the concept of online teaching is relatively weak. In the face of sudden online teaching, problems emerge, such as how to arrange classes, how to carry out online teaching according to the plan, which online teaching platform to choose, and how to monitor the effect and quality of online teaching, which will lead to the decrease in user satisfaction. Therefore, in this study, the impact of COVID-19 on user satisfaction is not only in the context of COVID-19, but it is also concluded that the usability of the platform is the main factor affecting users’ satisfaction with the online education platform in a unique period.

### 6.3. Education Financing Methods

Based on the research of online education satisfaction during the COVID-19 pandemic, we found that the platform availability is the most essential factor that affects the user satisfaction; therefore, we suggest that the enterprise that develops the online education platform puts more money into the technology research and development of platform availability in order to provide users with more satisfaction of online teaching services. To solve this problem, online education platform enterprises can consider several financing ways to raise funds. Online education platforms can be divided into two categories according to whether they charge fees or not. Therefore, this paper discusses the financing of online education platforms from two aspects. For online education platforms that charge tuition fees, there exist literatures that examine education financing problem. For example, Barr [54] set out the core lessons of financing higher education derived from economic theory and compares them with lessons of experiences of various countries. Jacobs and Wijnbergen [55] showed that public equity financing of education coupled with the provision of income insurance was the optimal way to finance education when private markets failed due to adverse selection. For online education platforms that do not charge fees, enterprises can consider the following ways of financing: (1) Enterprises can give priority to internal financing and rely on internal accumulation for financing, which includes three forms: capital, replacement investment converted by depreciation funds and new investment converted by retained earnings. Compared with external financing, internal financing can reduce information asymmetry and related incentive problems. (2) The enterprise may consider the way of introducing investment and seeking partner investment. During the COVID-19 pandemic, almost all college teaching activities have been transferred to online teaching. Enterprises can provide more detailed online teaching services to some well-known colleges to improve their online teaching quality. Hence, enterprises can find college partners to attract investment from colleges and universities. A practical example of this is as follows: Baidu Lecture Transfer reached a cooperation agreement with Georgia Institute of Technology, one of the three major American universities of science and Technology. Both sides will carry out in-depth cooperation in course introduction, technical research and joint exploration of an online education mode. (3) Enterprises can consider debt financing and obtain sufficient funds to research and develop technologies to improve the availability of online education platforms by issuing bonds or borrowing money for investments.

### 6.4. Internationalization of Conclusions

The analysis in this paper is based on the user data of Chinese online education platforms during the COVID-19 situation. In order to show that the conclusion of this paper is international, this part discusses whether the research results of this paper can be extended to other countries.

First of all, this paper finds that platform availability is the most important factor affecting the satisfaction of the online education platform, while the user personal factors have no significant impact on the satisfaction. In view of the similar online teaching methods adopted by countries all over the world, most of them adopt the forms of video conferences or live broadcasts. Therefore, the above conclusions can be extended to all countries in the world.

Secondly, this paper obtains the satisfaction evaluation system and satisfaction prediction model of an online education platform during the COVID-19 pandemic. Because this form of online education is still used in other countries, the evaluation system and prediction model obtained in this paper can be applied to the countries that have not yet recovered offline teaching. As long as relevant data are available, the BP neural network prediction model can be used.

## 7. Conclusions

This study collected user experience data on online education platforms in China during the COVID-19pandemic. Through emotion data analysis of online user reviews, we concluded that Ding Ding and Tencent Class provided high quality service, while Chaoxing Learning and MOOC encountered several problems, such as the inability to submit the learning time, lags, and a significant video delay. We extracted the factors influencing satisfaction and established a scientific and effective satisfaction index system using the existing literature. In addition, the data obtained from an offline questionnaire were examined and analyzed, and a structural equation model was built for quantitative analysis of the relationship between various indicators. It was found that users’ personal factors had no direct impact on their satisfaction, while platform availability had the greatest impact on user satisfaction. In addition, a BP neural network model was used to predict user satisfaction with online education platforms, and the prediction accuracy reached 77.5%. The model is thus highly effective.

Based on the above analysis and research, this paper proposes the following suggestions, which are expected to improve user satisfaction with online education platforms during public health emergencies:

(1) Platform technology problems cannot be ignored.
  Although an online education platform provides available teaching methods, there are still many problems in the platform technology. The design environment of the platform should be more concise and easy to operate, and the development of a dark mode is recommended. In terms of platform adaptability, there is the problem that mobile devices cannot switch between horizontal and vertical orientations. In terms of platform privacy, user authorization is required to control the user’s camera and microphone. Currently, there is a lack of platform customer service, and it is thus impossible to obtain timely feedback for problems with the platform. In terms of the platform function, the head portrait cannot be modified, the video progress bar cannot be synchronized, and other problems must be improved. In terms of the network technology environment, some platforms frequently encounter problems such as internet lags and network congestion. Technical problems in these platforms are the main factors affecting user experience that lead to students’ dissatisfaction and significantly reduce the efficiency and quality of teaching. Therefore, improving the platform technology is the primary problem to be solved.
  As for the above technical problems of the online education platform, the enterprises that belong to the platform should improve these problems, increase the investment in the online education platform and develop the function of the online system. Companies can obtain education financing through online crowd funding, initial coin offerings and other means, and use these financing methods to improve the quality and availability of online systems. As a result, these online education systems used in China can be extended to the world, increasing the use of the platform.
(2) The two-way interaction of teaching must be improved.
  Using a questionnaire of offline users, we analyzed eight first-level indicators selected by the structural equation, and determined that the main factors influencing user satisfaction with the online teaching platforms were system quality, interaction quality, service quality, and platform availability. Interaction environment refers to the effective communication environment in the process of knowledge acquisition. An increase in interaction can improve students’ learning enthusiasm and concentration. In a traditional classroom, there are various teaching interaction modes, such as reversed classroom, random questions, and group reports. However, in a network environment, the platform has few settings for teaching interaction, and teachers’ input teaching is the main teaching mode. Therefore, the platform must actively develop various interactive formats, such as “you ask me to answer”, “face to face”, “students record learning videos” and “real-time lecture” to promote efficient learning and further improve the quality of education.

## 8. Future Works

However, this paper still has the following limitations, which require further research:

(1) This paper only studies the satisfaction of online learning platforms from the perspective of students. In fact, the opinions of teachers and parents are also impactful. Therefore, future studies can comprehensively analyze the satisfaction of online education platforms from the perspective of multiple subjects.
(2) In this paper, the structural equation is used to predict the user satisfaction of the online learning platform, and the validity of the model is proved through analysis and verification. However, the questionnaire design and algorithm prediction need to be further improved.

## Figures and Tables

**Table 1:** Rank of teaching platform.

Teaching Platform	Platform List	Classification Ranking	Keyword Coverage	Total Scores
Ding Ding	1	1(General list)	18.799	1,730,000
Tencent Meeting	2	2(General list)	8526	382,000
Tencent Class	1	1(Education)	10.276	167,000
Chaoxing Learning	7	7(Education)	2043	437,000
Chinese MOOC	13	13(Education)	7031	818,000

**Table 2:** Questionnaire.

Classification of Investigation	Content of Investigation
User’s behavior on network teaching platform	Usage intention, device, learning effect, learning content
User experience	Degree of satisfaction, interactivity, platform availability, perceived value and so on
Basic information	Age, gender, education background

**Table 3:** Questionnaire data reliability information.

Index	Cronbach’s *α* Coefficient	Number of Questions
Degree of satisfaction	0.712	3
Intention of continuous use	0.771	2
Quality of interaction	0.781	3
Quality of service	0.751	4
Platform availability	0.786	4
Personal factors of users	0.727	5

Validity Test

**Table 4:** Data validity test.

Index	KMO	Bartlett Test of Sphericity
Degree of satisfaction	0.599	0.000
Intention of continuous use	0.500	0.000
Quality of interaction	0.500	0.000
Quality of service	0.500	0.000
Platform availability	0.765	0.000
Personal factors of users	0.500	0.000

**Table 5:** ROST CM (Regional Operations Support Team Content Mining) Emotional comments analysis.

Teaching Platform	Ding Ding	Tencent Meeting	Tencent Class	Chaoxing Learning	Chinese MOOC
Positive comments	6161	1703	1623	1289	471
Neutral comments	1915	43	558	28	30
Negative comments	3809	2531	1185	3628	2051

**Table 6:** NLPIR-Parser emotional scores

Teaching Platform	Ding Ding	Tencent Meeting	Tencent Class	Chaoxing Learning	Chinese MOOC
Total emotional score	10,454.5	870	933.5	−2190.5	−642
Positive score	56.319	7374	10,898.5	9022	1148
Negative score	45,864.5	−6504	−9965	−11,212.5	−1790

**Table 7:** Influencing factors.

Factor	Description
Platform Suitability	“computer”, “web”, “tablet”, “mobile terminal”, “incompatibility”
Platform privacy	“peep screen”, “prevention”, “call the camera”, “personal information”
Platform service type	“online customer service”, “staff service”
Platform teaching type	“recorded broadcast”, “live streaming”
Platform design environment	“blocking sight”, “simple”, “convenient and swift”, “dark mode”, “sharing the screen”, “main interface experience”, “interactive panel”
Platform functionality	“projection screen”, “horizontal screen”, “verification code”, “close microphone”, “vertical screen”, “rotating screen”, “scan a code”, “submit homework”, “self-rotating screen”
Network technology environment	“pop-up windows”, “network anomaly”, “bundled software”, “server exception”, “blank screen”, “load fail”, “system halted”, “network fluctuation”, “lost connection”

**Table 8:** Evaluation indicators affecting user satisfaction

The Primary Variable	The Secondary Variables	Indicators
User’s willingness to continue using	Recommend to others	Loy1—During the COVID-19 pandemic, target the online education platform you are satisfied with, you would like to recommend it to others
	Increase the frequency of use	Loy2—During the COVID-19 pandemic, the online teaching platform you are using will be used more in the future
User satisfaction	Learning needs	Sat2—During the COVID-19 pandemic, you think the existing functions of the online teaching platform can meet your learning needs
	Use feeling	Sat3—During the COVID-19 pandemic, you are very satisfied with the online teaching platform
	Attractive	Sat1—Compared with offline learning, you think online teaching during the COVID-19 pandemic is more attractive
Platform availability	Learnability	Pq2—During the COVID-19 pandemic, the steps of the online teaching platform you are using are easy to learn
	Easy to browse	Pq3—During the COVID-19 pandemic, the navigation system of online network teaching platform you use is clear, without confusion, and the page is easy to browse
	Interface design	Pq1—During the COVID-19 pandemic, the interface design of the online network teaching platform you are using is very reasonable
	Learning record	Pq4—During the COVID-19 pandemic, the online teaching platform you use can accurately record your learning time, learning content and learning information
The quality of interaction	Learner participation	Int1—During the COVID-19 pandemic, while learning online, you will actively answer the teacher’s questions and participate in the classroom learning
	Practice feedback	Int2—During the COVID-19 pandemic, you will complete the online study assignment assigned by the teacher on time
Information quality	Accuracy	A1—During the COVID-19 pandemic, which of the following difficulties and problems have you encountered while studying online? A2—During the COVID-19 pandemic, according to your common online teaching platform, what are the main ways to learn online? A3—During the COVID-19 pandemic, in the course of online teaching, what online interactions did you mainly participate in? A5—During the COVID-19 pandemic, which terminal can the online teaching platform you are using support for online learning?
	Integrity	
	Timeliness	
	Completeness	
System quality	High concurrent access	
	Security	
	Stability	
	Responsiveness	
The quality of service	Course management	Cq2—During the COVID-19 pandemic, the online teaching platform you use can recommend relevant courses according to what you watch
	Artificial service	Cq1—During the COVID-19 pandemic, when the online teaching platform fails, the customer service will help you to solve the problem in time
User personal factors	Education level	Per1—What kind of student are you?
	Use frequency	Per2—How often did you use an online teaching platform before COVID-19?
	Satisfaction tendency	Per3—When you use the online teaching platform for the first time, you will hold a completely negative attitude towards the platform because of some dissatisfaction with the use of the platform (such as registration trouble, slow login, etc.)
	Platform choice	A6—What platforms will you use as learning aids during and after the COVID-19 pandemic?

**Table 9:** Parameter analysis results.

Influence Elements	Path Coefficient	Influence Elements	Estimate	S.E.	C.R.	*p*
User satisfaction	<---	The quality of interaction	1.000		1.989	***
User satisfaction	<---	The quality of service	0.389	0.187	2.078	***
User satisfaction	<---	Platform availability	−0.236	0.224	2.032	***
User satisfaction	<---	User personal factors	0.760	0.382	0.417	0.047
User’s willingness to continue using	<---	User satisfaction	1.000			
The quality of interaction	<-->	The quality of service	0.273	0.049	5.557	***
The quality of service	<-->	Platform availability	0.262	0.042	6.225	***
The quality of interaction	<-->	Platform availability	0.217	0.035	6.276	***
Platform availability	<-->	User personal factors	0.002	0.016	0.102	0.918
The quality of service	<-->	User personal factors	−0.083	0.033	−2.528	0.011
The quality of interaction	<-->	User personal factors	−0.066	0.025	−2.639	0.008

Note that *** reflects the significance level when *p* < 0.001.C.R is the abbreviation of critical ratio. S.E is the abbreviation of Standard Error. <--> reflects the influencing factors are correlated. <--- reflects a causal relationship between the influencing factors.

**Table 10:** Fitness degree of model.

Indicators		Judgment Standard	Revised Model Results
Absolute fitness index	*χ* ^2^	the smaller the better	127.452
	*χ*^2^/df	1–3	2.360
	GFI	>0.9 better fit >0.8 can accept	0.938
	RMR	<0.08	0.054
	RMSEA	<0.08	0.066
Value-added fitness index	NFI	>0.9 better fit >0.8 can accept	0.914
	TLI	>0.9	0.925
	CFI	>0.9	0.948
Simple fitness index	PCFI	>0.5	0.633
	PNFI	>0.5	0.656

Note that *χ*^2^/df is the abbreviation of degree of freedom ratio; GFI: goodness-of-fit index; RMR: Root Mean Residual; RMSEA: Root Mean Square Error of Approximation, NFI: Normed fit index; TLI: Nonstandard fitting index; CFI: comparative fit index; PCFI: Simple adjustment comparison fit index; PNFI: parsimonious normed fit index.

**Table 11:** Path coefficients of the affecting factors.

Influence Elements	Path Coefficient	Influence Elements	Affect the Path
User satisfaction	<---	The quality of interaction	0.238
User satisfaction	<---	The quality of service	0.329
User satisfaction	<---	Platform availability	0.703
Pq2	<---	Platform availability	0.41
User’s willingness to continue using	<---	User satisfaction	1.000
Int2	<---	The quality of interaction	0.35
Int1	<---	The quality of interaction	0.10
Cq2	<---	The quality of service	0.5
Cq1	<---	The quality of service	0.4
Pq4	<---	Platform availability	0.34
Pq3	<---	Platform availability	0.33
Pq1	<---	Platform availability	0.63
Sat3	<---	User satisfaction	0.13
Sat2	<---	User satisfaction	0.64
Sat1	<---	User satisfaction	0.55
Loy1	<---	User’s willingness to continue using	0.55
Loy2	<---	User’s willingness to continue using	0.78
The quality of interaction	<-->	The quality of service	0.273
The quality of service	<-->	Platform availability	0.262
The quality of interaction	<-->	Platform availability	0.217

Note that <--> reflects the influencing factors are correlated. <--- reflects a causal relationship between the influencing factors.

**Table 12:** Division of satisfaction level.

Satisfaction Level	Descriptive	Divide the Scale
1	Very dissatisfied	*x ≤* 1.5
2	Dissatisfied	1.5 ≤ *x ≤* 2.5
3	General	2.5 ≤ *x ≤* 3.5
4	Satisfied	3.5 ≤ *x ≤* 4.5
5	Very satisfied	*x* ≥ 4.5

**Table 13:** Error indicator data.

Error Indicator	MSE	MAPE	RMSE	MSPE	SSE
Result	0.225	0.08	2.1213	0.55864	9

MAPE: Mean Absolute Percentage Error; RMSE: Root Mean Square Error; MSPE: Pure mean square error; SSE: The sum of squares due to error.
